# Human Embryonic Stem Cells Express Elevated Levels of Multiple Pro-Apoptotic BCL-2 Family Members

**DOI:** 10.1371/journal.pone.0028530

**Published:** 2011-12-09

**Authors:** David T. Madden, Diana Davila-Kruger, Simon Melov, Dale E. Bredesen

**Affiliations:** 1 College of Pharmacy, Touro University – California, Vallejo, California, United States of America; 2 Buck Institute for Research on Aging, Novato, California, United States of America; 3 University of California, San Francisco, San Francisco, California, United States of America; University of Sao Paulo – USP, Brazil

## Abstract

Two of the greatest challenges in regenerative medicine today remain (1) the ability to culture human embryonic stem cells (hESCs) at a scale sufficient to satisfy clinical demand and (2) the ability to eliminate teratoma-forming cells from preparations of cells with clinically desirable phenotypes. Understanding the pathways governing apoptosis in hESCs may provide a means to address these issues. Limiting apoptosis could aid scaling efforts, whereas triggering selective apoptosis in hESCs could eliminate unwanted teratoma-forming cells. We focus here on the BCL-2 family of proteins, which regulate mitochondrial-dependent apoptosis. We used quantitative PCR to compare the steady-state expression profile of all human BCL-2 family members in hESCs with that of human primary cells from various origins and two cancer lines. Our findings indicate that hESCs express elevated levels of the pro-apoptotic BH3-only BCL-2 family members NOXA, BIK, BIM, BMF and PUMA when compared with differentiated cells and cancer cells. However, compensatory expression of pro-survival BCL-2 family members in hESCs was not observed, suggesting a possible explanation for the elevated rates of apoptosis observed in proliferating hESC cultures, as well as a mechanism that could be exploited to limit hESC-derived neoplasms.

## Introduction

Apoptosis is a sophisticated mechanism for eliminating unwanted cells. The signaling pathways that regulate apoptosis vary among different cell types [1], [2], suggesting that apoptotic regulatory pathways are determined by differentiation status, wherein one cell lineage responds to apoptotic cues differently than others. Little is currently known about how the common precursor from which all tissues are derived - human embryonic stem cells (hESCs) - regulate entry into apoptosis. The importance of understanding these pathways is highlighted by one of the most significant obstacles to regenerative medicine: Transplantation of desired cell types contaminated with pluripotent cells can result in the formation of teratomas - tumors, usually benign, harboring differentiated cells of all lineages. If the primary pathways that govern apoptosis were to be determined in hESCs, strategies could be devised to exploit these pathways to eliminate potential teratoma-forming cells. Additionally, since large-scale expansion of hESCs remains a challenge, optimization of growth conditions could be achieved through reducing levels of apoptosis.

The most common apoptotic pathways are intrinsic pathways mediated via the mitochondrion [3], [4]. Varied cell death triggers cause mitochondrial outer-membrane permeabilization (MOMP), prompting release of cytochrome c from the mitochondrial inter-membranous space. Cytochrome c then activates caspases that effect destruction of the cell [3], [4]. MOMP is controlled by the BCL-2 protein family, which includes both pro-apoptotic (BAX and BAK) and pro-survival family members (BCL-2, BCL-xL, BCL-w, A1, and MCL-1), as well as the BCL-2 homology domain 3 (BH3)-only family members (BID, BAD, BIM, BIK, BLK, PUMA, NOXA, BNIP3, and HRK) [5]. The ultimate determinant of cell survival or apoptosis is the balance of active pro-survival BCL-2 family members and pro-apoptotic BCL-2 family members [5]. Not all BCL-2 family members are expressed in every cell type, and different triggers of apoptosis both activate specific pro-apoptotic BCL-2 family members and inactivate specific pro-survival BCL-2 family members [6], [7].

Considering their central importance in regulating apoptosis, determining the relative expression levels of the pro-apoptotic and pro-survival members of the BCL-2 family is an essential first step in describing apoptotic pathways in hESCs. In the current studies, we have addressed the following questions: (1) What is the expression of the compendium of BCL-2 family members in hESCs? (2) How does this gene expression profile compare to that in differentiated cell types? We compared the BCL-2 family member gene expression profile in pluripotent hESC lines TE06 and BG01 with those in hESC-derived neural stem cells, seven human primary cell lines from various origins, and two cancer cell lines (Table 1). The gene expression of five pro-survival BCL-2 family members, eight BH3-only BCL-2 family members, as well as BAX and BAK, was determined by quantitative reverse transcriptase polymerase chain reaction (qPCR).

**Table 1:** Cells used in this study.

Abbreviation	Cell Line Description	Source
TE06 (MEF)	hESC line TE06 co-cultured with MEFs	Xianmin Zeng^1^
TE06 (MAT)	hESC line TE06 grown on Matrigel in MEF-conditioned medium	Xianmin Zeng^1^
BG01 (MEF)	hESC line BG01 co-cultured with MEFs	NSCB^2^
BG01 (MAT)	hESC line BG01 grown on Matrigel in MEF-conditioned medium	NSCB^2^
TE06-NSCs	TE06-derived neural stem cells (NSCs)	This study
HDF	Dermal fibroblasts^3^	Invitrogen
HEK	Epidermal keratinocytes^3^	Invitrogen
HEMNn-LP	Epidermal melanocytes, neonatal, low pigmentation^3^	Invitrogen
HMVEC	Microvascular endothelial cells^3^	Invitrogen
HUVEC	Umbilical vein endothelial cells^3^	Invitrogen
HPASMC	Pulmonary artery smooth muscle cells^3^	Invitrogen
HMEC	Mammary epithelial cells^3^	Invitrogen
MCF-7	Human cancer cell line MCF-7	ATCC
HeLa	Human cervix Aden carcinoma cell line	ATCC

1 Buck Institute for Research on Aging, Novato, CA.

2 National Stem Cell Bank, WiCell Research Institute, Madison, WI.

3 Human primary cells.

We found that, compared to differentiated cells, proliferating hESCs express significantly higher levels of pro-apoptotic BCL-2 family members, including NOXA, BIK, BIM, BMF, and PUMA. Furthermore, elevated levels of these pro-apoptotic transcripts were not countered by elevated levels of pro-survival BCL-2 family members in hESCs. The results of this study suggest that hESCs might utilize unique mechanisms to regulate programmed cell death - mechanisms that could either be minimized to make large-scale production of hESCs more feasible or exploited to minimize the risk of teratoma formation upon transplantation of hESC-derived cell types.

## Results

In order to determine which BCL2 family members are expressed in pluripotent human embryonic stem cells, we compared the expression profile of BCL-2 family members between hESCs and differentiated cells (see Table 1) by qPCR.

### Selection of housekeeping genes

Because we chose to study gene expression in a collection of cells of seemingly disparate phenotypes, it was important to determine carefully the most appropriate reference gene(s) for normalization of the qPCR expression data. We followed the method described by Vandesompele et al. [8], which allows systematic evaluation of a collection of reference genes for those that vary least across the cell lines being analyzed. In this method, the normalization factor is a calculated value determined by the geometric mean of the expression levels of the best-performing reference genes (see methods, and REF [8]).

**Figure 1:**
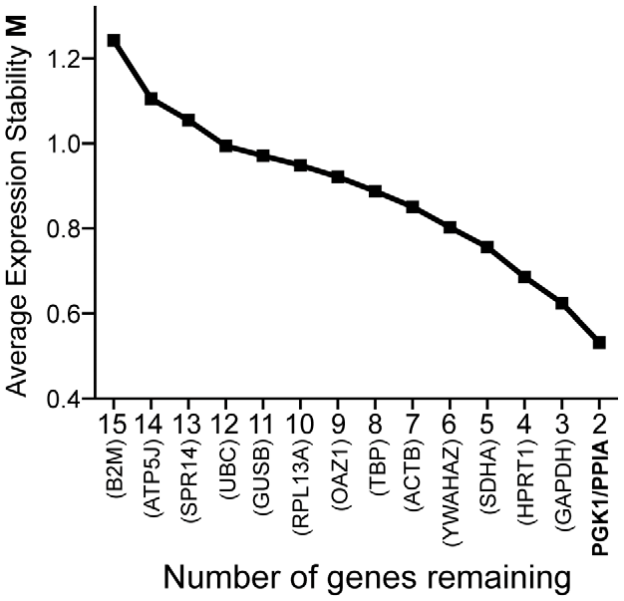
Selection of optimal reference genes: PGK1 and PPIA. 15 genes were evaluated for their appropriateness for use as reference genes to normalize all subsequent data. Using a method described by Vandesompele et al. [8] genes were ranked by a stability coefficient *M* and those genes having the lowest stability (greatest value of *M*) were eliminated from the subsequent round of stability calculations. The gene eliminated from each round is indicated in parentheses.

We selected a total of 15 genes for the purpose of normalizing qPCR data based their frequent use to normalize expression data (B2M, UBC, TBP, ACTB, YWHAZ, SDHA, RPL13A, GAPDH, HPRT1) or based on their identification as the least variant mRNAs among a large collection of gene expression data (ATP5J, SPR14, GUSB, OAZ1, PGK1, PPIA [9]). The expression stability among the set of cells outlined above, *M* (see materials and methods for details), was calculated for each gene from our data set. From this analysis, the least stable control genes were eliminated, and new values for *M* could be calculated from the remaining list of genes. The best-performing pair of genes, PGK1 and PPIA, was identified after step-wise exclusion of the least stable gene (highest value of *M*) in each of 13 iterative rounds of expression stability calculations (Figure 1).

### Expression of lineage-specific markers

**Figure 2:**
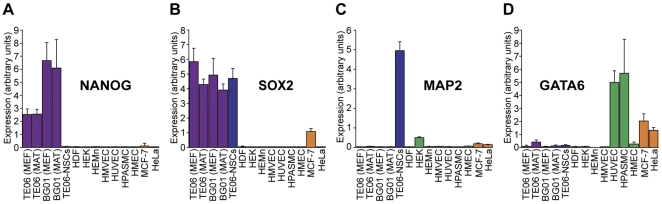
Cell lines express appropriate markers of pluripotency and lineage. Abundance of gene transcripts were determined by qPCR, and is expressed in linear arbitrary units. **A.** Expression of NANOG, a marker of pluripotency. **B.** Expression of SOX2, a marker of pluripotency and neural precursor cells. **C.** Expression of the microtubule-associated protein MAP2, a marker of neural cells. **D.** Expression of the transcription factor GATA6, a marker of heart and endoderm-derived tissues.

**Figure 3:**
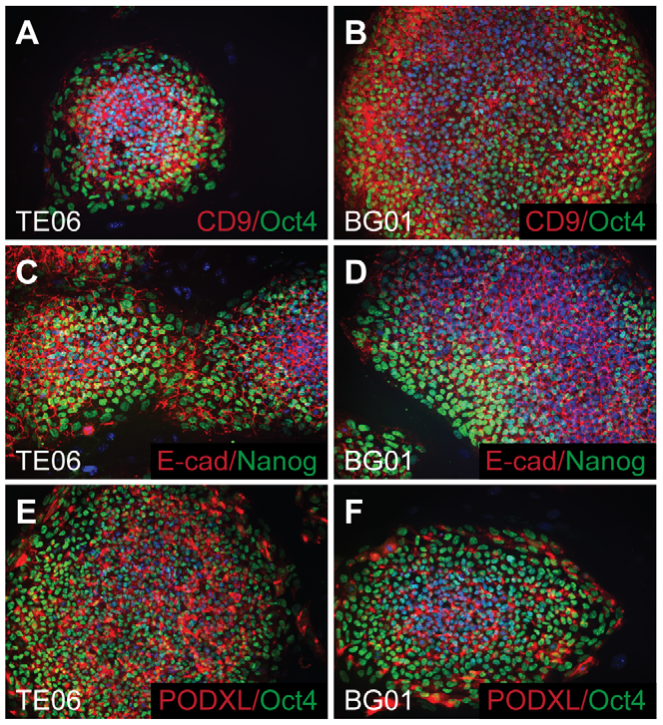
hESC lines BG01 and TE06 express protein markers of pluripotency. Antibodies against protein markers of pluripotency were used to label hESCs via immunofluorescence. Fixed TE06 (A, C, E) or BG01 (B, D, F) cells were incubated with primary antibodies directed against CD9 (A, B), Oct4 (A, B, E, F), E-cadherin (C, D), Nanog (C, D), or PODXL (E, F) followed by incubation with Alexa fluor 488 or 555 conjugated secondary antibodies. Pictures are 100× wide-field images captured on an inverted fluorescence microscope.

Once the optimum pair of reference genes was identified, we assayed the abundance of lineage-specific markers by qPCR. Expression of the transcription factors NANOG and SOX2 was primarily restricted to the hESC lines BG01 and TE06. Culturing cells with MEFs or on Matrigel in the presence of MEF-conditioned medium yielded similar expression (Figure 2). Additionally, the BG01 and TE06 cells also expressed pluripotency marker proteins E-cadherin, Oct-3/4, CD9, NANOG, and PODXL (Figure 3), as well as SOX2 (data not shown). SOX2 transcript was also elevated in TE06-derived neural stem cells (TE06-NSCs), consistent with the transcription profile of NSCs [10], [11].

We also tested the expression status of genes normally active in differentiated cells. Elevated transcript levels of MAP2, a neuronal marker, was seen only in TE06-NSCs (Figure 2). We also found that GATA-6, a transcription factor expressed in endoderm and some mesoderm-derived tissues, was predominantly expressed in pulmonary artery smooth muscle cells (Figure 2, [12]), but was also expressed in umbilical vein endothelial cells, consistent with what has been observed previously [13], [14]. Some GATA-6 expression could also be seen in the cancer lines MCF-7 and HeLa.

**Figure 4:**
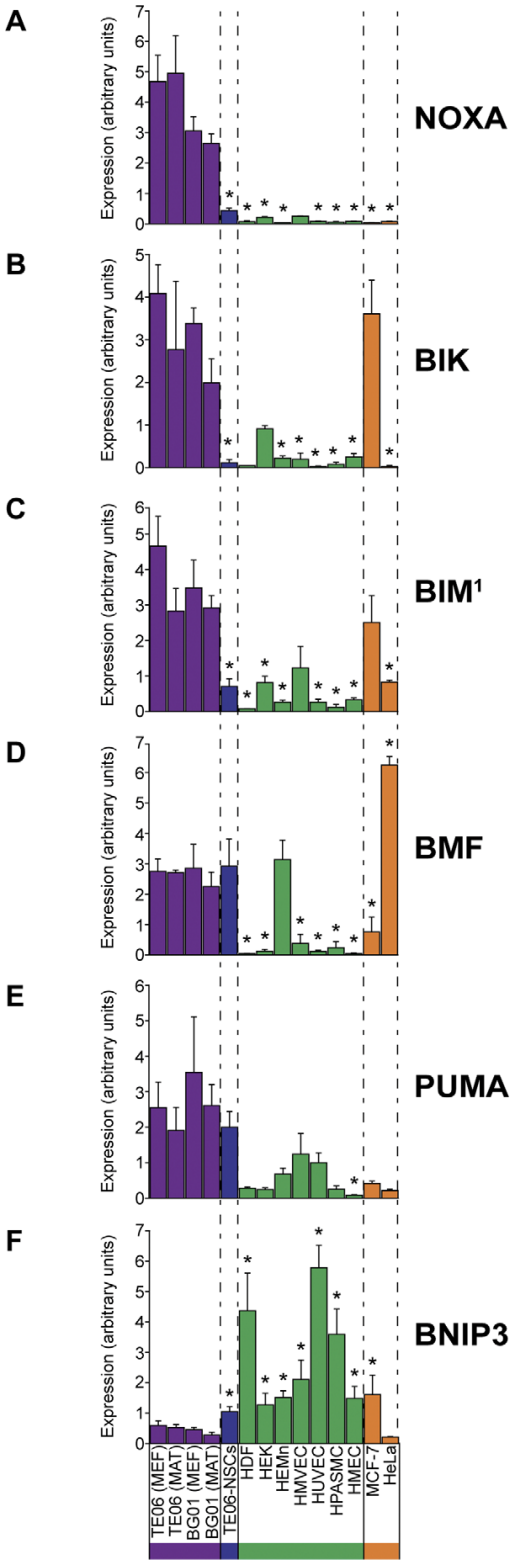
A cohort of pro-apoptotic BH3-only BCL-2 family members are expressed primarily in hESCs. Abundance of gene transcripts were determined by qPCR, and is expressed in arbitrary units. **A.** NOXA, **B.** BIK, **C.** BIM (assay detected all three transcript variants: BIM-L, BIM-EL, and BIM-S), **D.** BMF, **E.** PUMA, **F.** BNIP3. Asterisks indicate values that differ significantly from the average hESC expression level (*p<0.001*, two-tailed student's t test).

**Figure 5:**
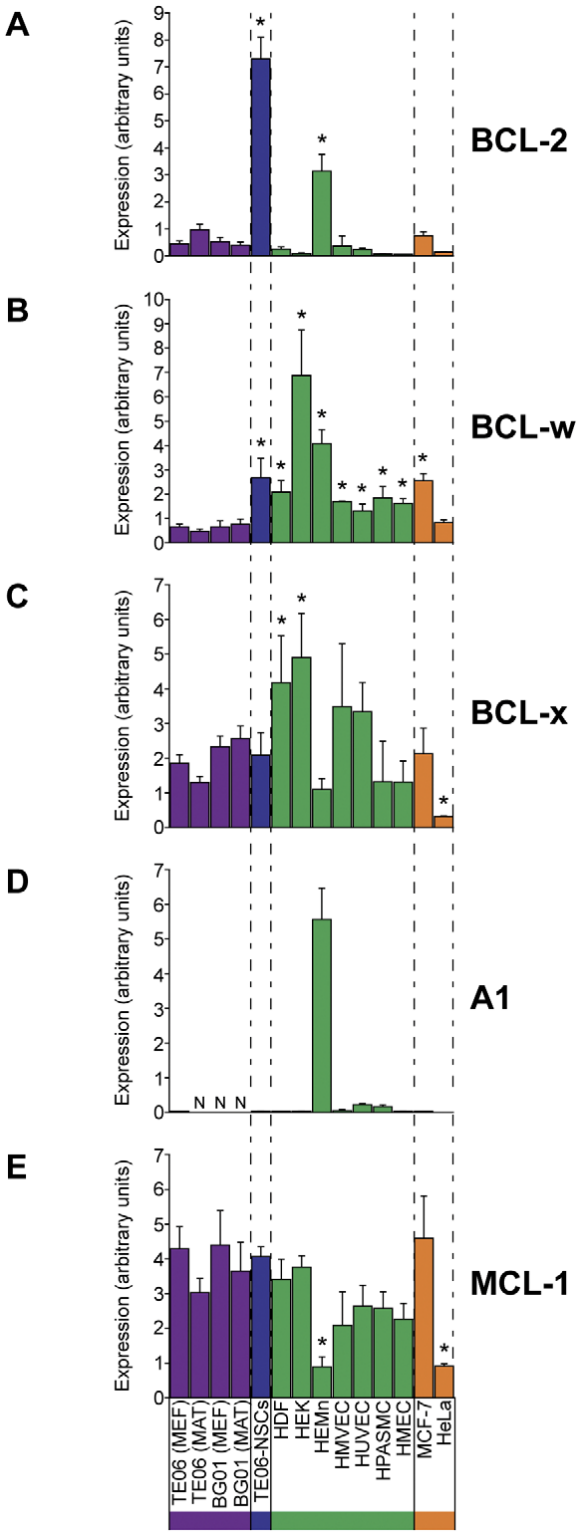
Expression of pro-survival BCL-2 family members. Abundance of gene transcripts were determined by qPCR, and is expressed in linear arbitrary units. **A.** BCL-2, **B.** BCL-w, **C.** BCL-x (assay detected both transcript variants: BCL-xS and BCL-xL), **D.** A1, **E.** MCL-1. Asterisks indicate values that differ significantly from the average hESC expression level (*p<0.001*, two-tailed student's t test).

The immediate goal of this study was to characterize BCL-2 family members in hESCs, and determine whether their expression was either unique or absent in pluripotent human embryonic stem cells. The method we chose to identify such BCL-2 members from the qPCR data was to calculate a ratio of the average BCL-2 family member expression in hESCs to the expression among the non-hES cell types. The median was used for the non-hES cell types because in many instances expression was particularly high, or low, in only one or two cell types. A simple ranking of the ratios for each BCL-2 family member revealed a surprising finding: The top five ranking BCL-2 family members having the greatest ratio of hESC:non-hESC expression were all pro-apoptotic BH3-only BCL-2 family members (Table 2). Two of the pro-apoptotic BCL-2 family members were expressed with a greater than 10-fold increase in hESCs versus non-hESCs: NOXA was expressed at an hESC:non-hESC ratio of 50, and BIK was expressed at an hESC:non-hESC ratio of 25 (Table 2). NOXA, BIK, BIM, and BMF were expressed in hESCs at levels that were significantly higher than those of the non-hES cell types (Figure 4, *p<0.001*, 2-tailed student's t test, for most pair-wise comparisons between non-hES cell type and average hESC expression). Not every pro-apoptotic BCL-2 family member transcript was elevated in hESCs versus non-hES cell types, however: The pro-apoptotic BH3-only family member BNIP3 was ranked second only to the pro-survival BCL-2 family member A1 in the non-hESC:hESC expression ratio (Table 2 and Figure 5).

**Table 2:** BCL-2 family members with hESC or non-hESC expression bias.

	hESC^1^:Non-hESC^2^		Non-hESC^2^:hESC^1^	
Rank	Gene	Ratio	Gene	Ratio
1	NOXA	50.1	A1	102
2	BIK	25.8	BNIP3	3.3
3	BIM^3^	8.1	BCL-w	3.1
4	BMF	8.0	BAD	1.9
5	PUMA	7.4	BID	1.2
6	BIM^4^	4.7		
7	BCL2	3.7		

1 Mean expression of all hESC samples.

2 Median of non-hESC expression values.

3 qPCR assay covers all three BIM transcript variants: BIM-L, BIM-EL, and BIM-S.

4 Different primer/ probe set that also detects all three BIM transcripts.

We expected to find instances wherein anti-apoptotic BCL-2 family members would be elevated in hESCs relative to the other cell types, so as to counter the hESC-restricted activity of the pro-apoptotic BH3-only BCL-2 family members NOXA, BIK, BIM, BMF, and PUMA. However, none of the five pro-survival BCL-2 family members we assayed demonstrated hESC-specific expression to the extent that NOXA, BIK, BMF, and PUMA did. Pro-survival BCL-2 family members were found to have either greater expression in non-hES cell types than hESCs (A1 and BCL-w) or statistically insignificant differences between hESCs and non-hESCs (BCL-x and MCL-1). The one exception was the canonical anti-apoptotic molecule, BCL-2, whose average expression in hESCs was about 4-fold greater than the median non-hESC expression.

As an independent means of identifying BCL-2 family members that are uniquely expressed in either hESCs or non-hESCs, we used a clustering alignment algorithm to group the genes based on expression profiles. We included in our clustering analysis genes that are markers of pluripotency (SOX2 and NANOG) as well as markers of differentiated cells (MAP2 and GATA6). We also included the tumor suppressor p53 in our clustering analysis based on our finding that of the top five ranked genes having the greatest hESC-specific expression pattern (Table 2), three of these are direct targets of p53 transactivation activity: NOXA [15], BIK [16], and PUMA [17], [18]. BAX, another known target of p53 [19], demonstrated slightly less hESC-specific expression, ranking seventh on a list of all genes from this study. Furthermore, recent evidence suggests that BIM is an indirect target or p53 [20].

**Figure 6:**
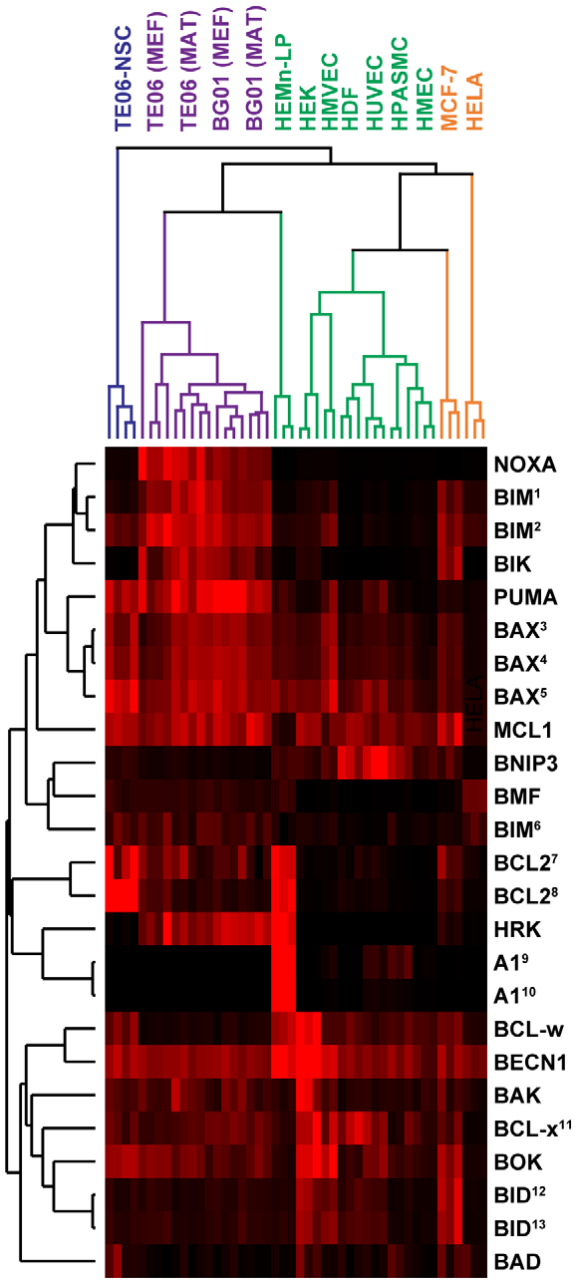
Hierarchical cluster analysis of BCL-2 family members in hESCs and non-hESCs. qPCR data was clustered via a two-way unsupervised clustering algorithm (Cluster 3.0) and visualized as a heat map (Java TreeView 1.1). Parallel cultures of each cell line (number): TE06-NSCs (4), TE06 MAT (4), TE06 MEF (4), BG01 MEF (4), BG01 MAT (4), HEMn-LP (3), HEK (3), HMVEC (2), HDF (3), HUVEC (3), HPASMC (3), HMEC (3), MCF-7 (3), and HeLa (3). For many genes, assays detected all or a subset of the transcript variants. ^1^Variants 1–7. ^2^Variants alpha, beta, delta, and sigma. ^3^Variants alpha and delta. ^4^Varient sigma. ^5^BIM-L, BIM-EL, and BIM-S. ^6^BIM-L, BIM-S. ^7^BIM-S. ^8^Variant 5 ^9^Variant alpha. ^10^Variants alpha and beta. ^11^Variant 1. ^12^Variant 2. ^13^BCL-xL and BCL-xS. ^14^Variant 3. ^15^Variants 1 and 2.

We found that p53-regulated pro-apoptotic BCL-2 family members were clustered into two groups having very similar expression profiles: One group included p53 itself, along with the pluripotency marker SOX2 and the pro-apoptotic BCL-2 family members PUMA and BAX (Figure 6, correlation coefficient 0.75). The other group consisted of the pluripotency marker NANOG as well as the pro-apoptotic BCL-2 family members BIK, NOXA, and BIM (Figure 6, correlation coefficient 0.65). Although other smaller clusters of gene expression profiles were observed, none were correlated to the same degree as those seen clustered with SOX2 and NANOG. These results, taken together with the BCL-2 comparison data, suggested that high basal transcript expression of potent pro-apoptotic BCL-2 family members PUMA, NOXA, BIM, BIK, and BAX, is a genuine general feature of pluripotent human embryonic stem cells.

## Discussion

In this study we show that, when compared to differentiated cells or cancer cells, hESCs express elevated levels of multiple pro-apoptotic BCL-2 family members. We took advantage of a microfluidics-based qPCR platform to measure all of the human BCL-2 family members across 48 samples simultaneously. The samples consisted of 12 cell lines each having 3 or, in some cases, 4 replicate cultures, which were established from separately thawed cryologically-preserved cell stocks. Additionally, hESC lines (BG01 and TE06) were each grown under both MEF feeder and feeder-free conditions. Owing to the disparate cell types evaluated for gene expression, we first measured expression levels of 15 reference genes, and identified two genes (PGK1/PPIA) that best reflected total mRNA levels. As a result of this experimental design, we are confident that the gene expression levels calculated from our qPCR experiments accurately reflect differences in abundance of mRNA transcripts among the dozen cell types studied. We hypothesized that one, or perhaps two BH3-only BCL-2 family members would be dominantly expressed in hESCs, and that these pro-apoptotic transcripts would be balanced by select pro-survival BCL-2 family members. From these patterns of expression, we hypothesized that the principal pathways governing apoptosis in hESCs could be revealed.

Contrary to our expectations, we found that hESCs express the pro-apoptotic BH3-only BCL-2 family members NOXA, BIK, BIM, BMF, and PUMA at levels far greater than that seen in the seven human primary cells, hESC-derived neural stem cells, or cancer lines (Figure 4). However, only one of the pro-survival BCL-2 family members, BCL-2, was found to be moderately elevated in hESCs compared to that seen in other cell types. Transcript abundance of two other pro-survival BCL-2 family members, BCL-x and MCL-1, was merely comparable to that seen in the other ten cell lines, and expression levels of BCL-w, and A1 were minimal in hESCs (our assays did not distinguish between transcripts for BCL-xL and BCL-xS). What is more, unsupervised hierarchical clustering of these gene expression profiles resulted in the clustering of NOXA, BIK, BIM, BMF, and PUMA together with hESC pluripotency markers like NANOG and SOX2. We interpreted these findings as further indication that elevated expression of these BH3-only BCL-2 family members is a genuine feature of hESCs when they are either co-cultured with MEFs or grown on Matrigel with conditioned media.

Despite the fact that well over a decade has passed since the first human embryonic stem cell lines were established, we have yet to uncover the complete list of MEF-derived molecular determinants of hESC pluripotency and proliferation [21], [22]. It is formally possible that a deficient trophic environment explains the ubiquitous observation that hESCs have elevated rates of apoptosis [23]–[25]. From this, it would logically follow that death of hESCs in culture might require certain pro-apoptotic BH3-only BCL-2 family members. However, it was only recently reported that BCL-2 family members play a role in governing basal rates of apoptosis in hESCs [23], [26]. In one study by Ardehali et al., BCL-2 overexpression in hESCs was found to improve overall growth rates, eliminate reliance on knock-out serum replacement, increase clonogenicity after single cell dissociation, and reduce apoptosis associated with newly formed embryoid bodies [26]. In another study by Ohgushi et al., apoptosis in hESCs resulting from single cell dissociation was shown to be inhibited by overexpression of the pro-survival BCL-2 family member BCL-xL [23]. That a pro-survival BCL-2 family member could reduce dissociation-induced apoptosis and increase the overall growth rate of hESCs suggests that basal rates of apoptosis are most likely mediated by constitutive expression of pro-apoptotic BH3-only family members. Our data suggest that the pro-apoptotic BH3-only family members responsible for basal rates of apoptosis include NOXA, BIK, BIM, BMF, and PUMA, or a subset thereof.

Despite the constitutive overexpression of the pro-apoptotic BCL-2 family members NOXA, BIK, BIM, BMF and PUMA in hESCs, it is conceivable that the activities of these molecules would be inhibited via post-translational modifications, and that apoptosis in hESCs may be controlled by other novel signaling pathways. Accumulating evidence in the literature indicates that many BH3-only BCL-2 family members are regulated by post-translational modifications [5]. Whereas nothing is currently known about what, if any, post-translational modifications of pro-apoptotic BH3-only BCL-2 family members might occur in hESCs, limiting the apoptotic activity of these molecules to some extent would be essential for net proliferation to occur.

For many of the pro-apoptotic BCL-2 family members, including BAD, BIM, BMF, BIK and NOXA, apoptosis-promoting activity can be regulated by phosphorylation. Perhaps the most well known example of this is in the case of BAD, wherein phosphorlyation of serine residues by multiple kinases, including AKT, can promote complex formation with 14-3-3, resulting in cytosolic sequestration [5], [27]–[39]. Similarly, ERK1/2 or RSK1/2-mediated phosphorylation of BIM blunts the pro-apoptotic activity of this molecule [5], [40], [41]. Instead of being sequestered in the cytosol by 14-3-3, as in the case of BAD, BMF associates with DLC2, thereby limiting BMF's pro-apoptotic activity [5], [42]. BIK is also under post-translational control, but in this case, casein kinase-mediated phosphorylation of BIK stimulates its apoptotic function [5], [43], [44]. More recently, NOXA was also discovered to be the target of phosphorylation [45]. The atypical cyclin-dependent kinase CDK5 phosphorylates NOXA when cells experience glucose replete conditions. Phosphorylation of NOXA causes cytosolic sequestration via an unknown mechanism and blunted apoptotic function, in addition to inducing a novel NOXA-mediated stimulation of the pentose phosphate pathway [45]. To date, no evidence of post-translational modifications of PUMA have been identified, but based on the growing list of post-translational modifications that regulate BCL-2 family member function, post-translational modifications of PUMA remain a distinct possibility.

The picture that emerges is one in which hESCs may express pro-apoptotic BH3-only BCL-2 family members in a constitutive fashion, but that the apoptotic function of these molecules could be held in check either by complex formation with pro-survival BCL-2 family members (likely BCL-xL or MCL-1) or by apoptotic-inhibitory phosphorylation of NOXA, BIK, BIM, BMF and possibly PUMA, or complex formation with other inhibitory molecules (e.g., 14-3-3 for BAD, DLC2 for BMF). The advantage of such a scenario might be that apoptotic responses could be engaged more rapidly and robustly, requiring only kinase cascades to control activation of apoptosis. The danger to the cell, and a possible explanation of the challenge of growing hESCs, is that seemingly minor changes in the environment could potentially result in cell death.

In conclusion, we have shown that, compared with differentiated cells, hESCs express an unusual repertoire of BCL-2 family members. hESCs cultured under standard growth conditions contain elevated levels of transcripts encoding pro-apoptotic BH3-only BCL-2 family members NOXA, BIK, BIM, BMF, and PUMA, when compared to differentiated cells. This, in light of the fact that complementary increases in pro-survival BCL-2 family members are not seen in hESCs, suggests that this expression profile could help to explain the elevated rates of apoptosis seen in hESCs. Recent reports demonstrating that overexpression of BCL-2 [26] or BCL-xL [23] limits dissociation-induced apoptosis and increases overall growth rates is compatible with our finding that hESCs express elevated levels of pro-apoptotic BH3-only BCL-2 family members, and thus may be “primed” for apoptosis in a way that differentiated cells are not, and supports the notion that improvements in hESC culture methods could reduce the basal rates of apoptosis. With the large-scale propagation of hESCs remaining a major hurdle to the development of hESC-based therapies, our ability to limit the basal rate of apoptosis will be an important step toward achieving this goal.

Furthermore, given that, even in the face of elevated expression of pro-apoptotic BH3-only BCL-2 family members, hESCs are able to proliferate under standard growth conditions, hESCs might utilize novel regulatory mechanisms to control initiation of apoptosis. The importance of such a possibility is highlighted by the fact that one of the greatest obstacles to overcome in regenerative medicine is the potential of introducing teratoma-forming cells during transplantation of cells with desired phenotypes. Therefore, our ability to exploit apoptotic pathways unique to hESCs could improve the clinical outcome of therapies relying on hESC-derived cells.

## Materials and Methods

### Ethics statement

All protocols involving hESCs were approved by the UC Davis Stem Cell Oversight Committee. The two human embryonic stem cell lines used in this study were TE06 and BG01. TE06 cells were received from the laboratory of Xianmin Zeng, Buck Institute for Research on Aging, Novato, CA. BG01 cells were purchased from the National Stem Cell Bank, WiCell Research Institute, Madison, WI.

### Growth of non-hESCs

HeLa cervical carcinoma cells and MCF-7 breast carcinoma cells were purchased from the American Type Culture Collection (ATCC, Manassas, VA), and were cultured in DMEM, 2 mM L-glutamine, 50 units/ml penicillin G, 50 ug/ml streptomycin sulfate (Life Technologies, Carlsbad, CA) and 10% FBS (Sigma, St. Louis, MO). The seven human primary cell lines used in this study were purchased and grown exactly as recommended by the manufacturer using specialty media all supplied by the manufacturer (Life Technologies). Human umbilical vein endothelial cells (HUVEC, Life Technologies) were grown in LSGS-supplemented Medium 200. Human epidermal melanocytes-neonatal lightly pigmented cells (HEMn-LP, Life Technologies) were grown in HMGS-supplemented Medium 254. Human mammary microvasculature endothelial cells, neonatal dermis (HMVECnd, Life Technologies) were grown in MVGS-supplemented Medium 131. Human pulmonary artery smooth muscle cells (HPASMC, Life Technologies) were grown in SMGS-supplemented Medium 231. Human epidermal keratinocytes, neonatal (HEKn, Life Technologies) were grown in HKGS-supplemented Epilife media. Human dermal fibroblasts, neonatal (HDFn, Life Technologies) were grown in LSGS-supplemented Medium 106. Human mammary epithelial cells (HMEC, Life Technologies) were grown in HuMEC- and bovine pituitary extract-supplemented HuMEC Basal Serum Free Medium.

### Growth of hESCs on Mouse Embryonic Fibroblasts

hESC lines BG01 and TE06 were propagated on mitomycin-C-inactivated MEFs (Millipore, Billerica, MA) in hESC medium: DMEM/F12 (1∶1) with 20% knockout serum replacement, 2 mM nonessential amino acids, 2 mM L-glutamine, 50 units/ml penicillin G, 50 ug/ml streptomycin sulfate (all from Life Technologies), 0.1 mM beta-mercaptoethanol (Millipore, Billerica, MA), and 4 ng/ml bFGF (PreproTech, Rocky Hill, NJ). hESCs were passaged enzymatically every 7 days. Briefly, cells were washed with DMEM/F12 (1∶1), then incubated with collagenase IV (1 mg/ml in DMEM/F12, Life Technologies) for 40 minutes at 37°C and 5% CO_2_. Any semi-adhered hESC colonies were pushed off the plate with a plastic pipette, and colonies were sedimented via centrifugation. Cell colonies were broken up into smaller cell aggregates by trituration of the harvested colonies in hESC medium. Triturated cells were then plated on new mitomycin-C-inactivated MEFs and incubated at 37°C with 5% CO_2_ for 48 h, and media was changed daily.

### Growth of hESCs without feeders

MEF conditioned medium (CM) was prepared by culturing mitomycin-C-inactivated MEFs in hESC medium. After 24 h of incubation, the medium was collected and stored at 4°C. The conditioned medium collected for six consecutive days from the same MEF culture was pooled, filtered through a 0.2 um membrane (Nalgene, Rochester, NY), and aliquots were stored at −20°C. Before use, thawed CM aliquots were supplemented with an additional 4 ng/ml bFGF. hESCs were harvested using collagenase as described above, with the exception that colonies were triturated in CM. The dissociated hESCs were seeded on Matrigel-coated plates (1∶50 dilution, BD, Franklin Lakes, NJ) in CM, and media was changed daily with CM until the hESCs reached approximately 65% confluency. Cells were passaged every 7 days.

### Immunocytochemistry

Immunostaining was performed as described in Madden et al. [46]. Briefly, hESCs were co-cultured with MEFs on glass coverslips coated with gelatin. Cells were fixed in 4% PFA for 10 min, permeabilized in 0.05% saponin for 5 min, and incubated in ice-cold acetone for 15 min. Primary antibodies (all from R&D Systems, Minneapolis, MN) were diluted with 1% wt/v BSA (Sigma) in PBS (PBA), and incubated with cells for 1.5 h at room temperature; anti-NANOG (R&D Systems cat no. AF1997, 1∶100), anti-PODXL (cat no. MAB1658, 1∶100), anti-SOX2 (cat no. MAB2018, 1∶100), anti-OCT3/4 (cat no. AF1759, 1∶100), anti-CD9 (cat no. MAB1880, 1∶100), and anti-E-Cadherin (cat no. MAB1838, 1∶10). Anti-mouse or anti-rabbit secondary antibodies conjugated to either Alexa Fluor 488 or Alexa Fluor 555 (Life Technologies) were diluted 1∶200 in PBA and incubated for 1.5 h at room temperature in the dark. Washed cells were then post-fixed with 2 ug/ml Hoechst 33342 (Life Technologies) in 4% PFA for 10 min and mounted on to glass slides with SlowFade antifade reagent following the manufacturer's instructions (Life Technologies). Fluorescence images were viewed with Nikon fluorescence objectives using a Nikon TE300 (Nikon Inc., Melville, NY). Images were captured with a Photometrics CoolSNAP EZ digital camera (Roper Scientific, Inc., Germany) using Simple PCI software (Compix Inc. Imaging Systems, Cranberry Township, PA). The brightness, contrast, and unsharp mask functions of Adobe Photoshop CS were used to optimize the images (Adobe Systems Inc., San Jose California).

### Karyotypic analysis

Karyotypic analysis of our expanded banks of hESCs (BG01 and TE06) was performed by Cell Line Genetics (Madison, WI). See Figure S1.

### RNA Isolation

For the purposes of this study, cultures were established from liquid nitrogen storage from three or, in some cases, four vials of a previously expanded cell line. Replicate cultures were maintained in parallel until sufficient quantities for RNA extraction were grown. Independently established cultures were never mixed or combined with other cultures of the same line.

RNA was isolated from cells grown in parallel cultures (replicate *n*): BG01 on MEFs (4), BG01 on Matrigel (4), TE06 on MEFs (4), TE06 on Matrigel (4), TE06-derived NSCs (4), human pulmonary smooth muscle cells (3), human mammary epithelial cells (3), human epidermal keratinocytes (3), human epidermal fibroblasts (3), human umbilical vein endothelial cells (3), human microvascular endothelial cells (3), human epidermal melanocytes (3), the cancer lines HeLa (3) and MCF7 (3). RNA was also isolated from mouse embryonic fibroblasts to verify that qPCR primer-assay pairs were human-specific.

hESC colonies were harvested in a similar fashion used for routine passaging with the exception that cells were treated with collagenase for 20 min only. Colonies were collected by centrifugation (200×g) for 2 min, the supernatant was aspirated, cells were frozen in a bath of dry-ice/ ethanol, and transferred to a −80°C cryo-freezer for storage. The seven primary cell lines (human pulmonary smooth muscle cells, human mammary epithelial cells, human epidermal keratinocytes, human epidermal fibroblasts, human umbilical vein endothelial cells, human microvascular endothelial cells, human epidermal melanocytes) as well as the two cancer lines (HeLa and MCF-7) were harvested by trypsinization, neutralized with trypsin inhibitor (primary cells only), collected by centrifugation, decanted, and frozen on dry-ice/ ethanol. Frozen pellets were transferred to a −80°C cryo-freezer for storage. After eight months of storage, RNA was isolated using Quiagen RNeasy kits (Quiagen, Valencia, CA).

### Microfluidic Polymerase Chain Reaction

We used a BioMark™ real-time PCR system (Fluidigm, San Francisco, CA, USA) in conjunction with a BioMark 48.48 Dynamic Array, which has high throughput capacity due to its integrated fluidic circuit feature [47]. First-strand cDNA was prepared using SuperScript III First-Strand Synthesis SuperMix for qPCR according to the manufacturer's recommendations (Life Technologies). Prior to analysis, cDNA from each sample was pre-amplified using a 0.2× mixture of 25 Universal Probe Library gene expression assays (Roche Applied Science, Indianapolis, IN) and TaqMan® PreAmp Master Mix (Life Technologies). cDNA (1.25 µl) was amplified in a reaction (5 µl) for 14 cycles according to the manufacturer's recommendations. At the end of thermal cycling, the reactions were diluted 1∶5 in low EDTA TE (10 mM Tris-HCl, pH 8.0; 0.1 mM EDTA, pH 8.0). Samples were prepared for loading into the dynamic array by mixing TaqMan® Universal Master Mix (2.5 µl, Life Technologies), DA Sample Loading Reagent (0.25 µl, PN 85000735; Fluidigm) and pre-amplified cDNA (2.25 µl). The 48.48 Dynamic Array (PN BMK-M-48.48; Fluidigm) was primed according to the manufacturer's recommendations. Samples (5 µl) were loaded into separate sample inlets on the Dynamic Array. Gene expression assays (Roche Applied Science) prepared as 20× solutions were diluted to 10× using the DA Assay Loading Reagent (PN 85000735; Fluidigm) and aliquots (5 µl) were loaded into separate reagent inlets on the Dynamic Array. Each assay was run as technical triplicates or quadruplicates, and loading positions across the array were randomized. The dynamic array was placed on a Nanoflex™ 4-IFC Controller (Fluidigm) for loading and mixing. After loading, the dynamic array was placed on the BioMark™ real-time PCR system for thermal cycling and real-time imaging of the reactions. Thermal cycling conditions were: 50°C for 2 min with AmpErase UNG (Life Technologies), 95°C for 10 min hot start, and 40 cycles of 95°C for 15 s and 60°C for 1 min. Ct values were determined using BioMark real-time PCR analysis software. All other calculations, including reference gene stability, were performed using Microsoft Excel (Microsoft, Redmond, WA).

We used the Universal Probe Library (Roche Applied Science, Indianapolis, IN) set of sequence-specific probes and relied on the Universal Probe Library assay design tool (www.roche-applied-science.com) to choose primer sequences and the appropriate fluorescent sequence-specific probe. All primers were purchased from Sigma (St Louis, MO). For the list of all primer and probe sequences used in this study, see Table S1.

### Data processing

C_t_ values were determined from the 47 samples based upon the mean of the C_t_s from technical replicates of assays used to detect 15 different putative reference genes. Next, the set of 47 C_t_ values was converted to relative quantity for each of the 15 gene assays. Calculations were performed as described by Vandesompele et al. [8]. Briefly, a new array *A_jk_* was calculated as the log_2_-transformed expression ratios *a_ij_/a_ik_* for each combination of two control genes *j* and *k* (*i* = 1 to *m* elements, *m* = 47 for this study, equation 1). The pairwise variation *V_jk_* was calculated as the standard deviation of the *A_jk_* elements (equation 2). The stability of the control gene *j* (*M_j_*) was defined as the arithmetic mean of all pairwise variations *V_jk_* (equation 3).
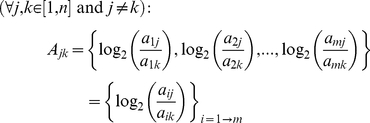
(1)

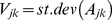
(2)

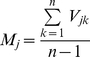
(3)The 15 reference genes were ranked in order of their stability *M*: The reference gene having the lowest gene stability (highest value of *M*, and in this case B2M) was eliminated from a new data set having 14 control genes, and new values of *A_jk_*, *V_jk_*, and *M_j_* were calculated for the new data set. This process was repeated iteratively until only two genes were remaining of the initial 15: PPIA and PGK1. The geometric mean of these two genes was used to normalize the expression of all other genes for each of the subsequent six dynamic arrays where expression levels of query genes were assayed.

### Clustering Analysis

Gene expression data were analyzed using the clustering analysis software Cluster (Stanford) based on a clustering algorithm described previously [48]. The similarity metric was absolute correlation (centered) using the average linkage clustering method. The clustered data was displayed as a heat map using Java TreeView [49].

## Supporting Information

Figure S1 **Karyotypic analysis of hESC lines BG01 and TE06.**
(TIF)

Table S1 **qPCR primer and UPL probe sequences used in this study.**
(XLS)
