# Correction: Human Embryonic Stem Cells Express Elevated Levels of Multiple Pro-Apoptotic BCL-2 Family Members

**DOI:** 10.1371/annotation/38d5bc7f-42f2-4d1e-b293-1a003bb57812

**Published:** 2012-09-12

**Authors:** David T. Madden, Diana Davila-Kruger, Simon Melov, Dale E. Bredesen

There was an error in Figure 6. The corrected image of Figure 6 can be seen here: 

**Figure:**
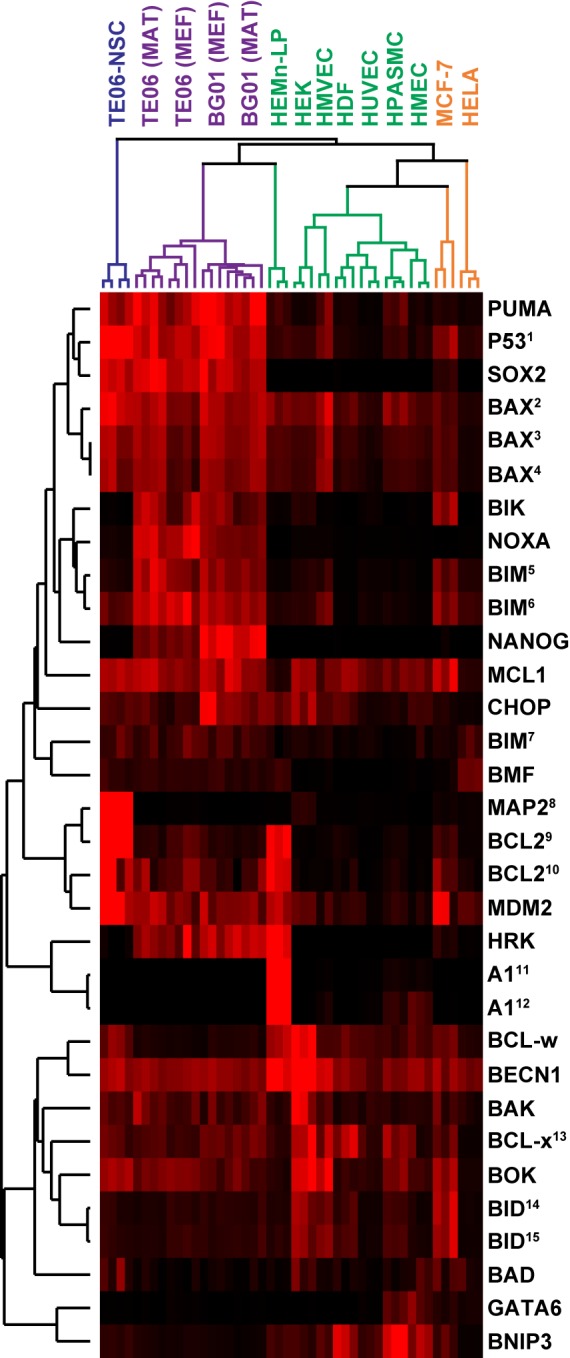


[^] 

