# Supplementary figures and images for: Human Embryonic Stem Cells Express Elevated Levels of Multiple Pro-Apoptotic BCL-2 Family Members
Source: PLoS One. 2011 Dec 9;6(12):e28530. doi: 10.1371/journal.pone.0028530 (PMC3235131; doi:10.1371/journal.pone.0028530)

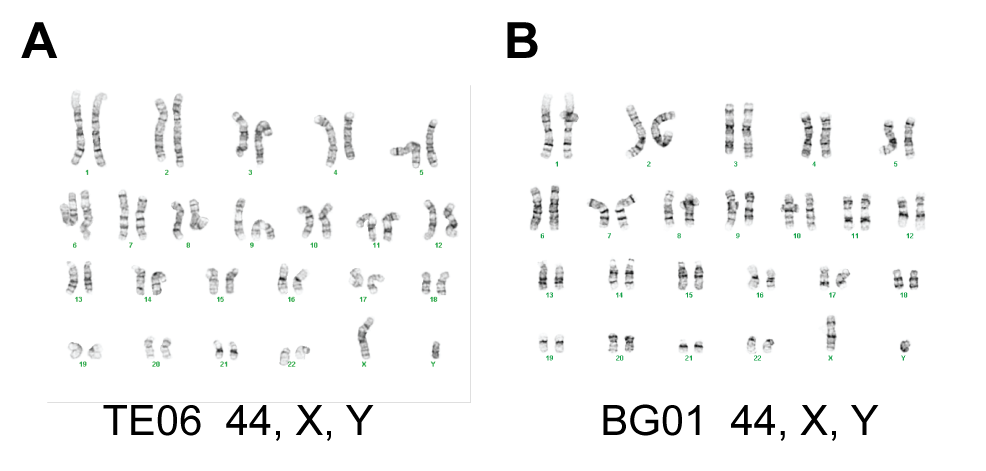

Supplement: Figure S1 — Karyotypic analysis of hESC lines BG01 and TE06. (TIF) [file pone.0028530.s001.tif]
